# Virtually spatialized sounds enhance auditory processing in healthy participants and patients with a disorder of consciousness

**DOI:** 10.1038/s41598-021-93151-6

**Published:** 2021-07-01

**Authors:** Lizette Heine, Alexandra Corneyllie, Florent Gobert, Jacques Luauté, Mathieu Lavandier, Fabien Perrin

**Affiliations:** 1 Audition Cognition and Psychoacoustics Team, Lyon Neuroscience Research Center, UCBL, INSERM U1028, CNRS UMR5292, Centre Hospitalier Le Vinatier, Bâtiment 462, Neurocampus Michel Jouvet, 95 Boulevard Pinel, Bron Cedex, 69675 Lyon, France; 2 Laboratoire de Tribologie et Dynamique des Systèmes UMR 5513, ENTPE, University of Lyon, Rue Maurice Audin, 69518 Vaulx-en-Velin Cedex, France; 3 Service de Médecine Physique et de Réadaptation, Rééducation Neurologique, Hôpital Henry-Gabrielle, CHU de Lyon, 69230 Saint-Genis-Laval, France; 4 Trajectoires Team, Lyon Neuroscience Research Center, UCBL, INSERM U1028, CNRS UMR5292, Centre Hospitalier Le Vinatier, Lyon, France

**Keywords:** Auditory system, Cognitive neuroscience, Sensory processing, Trauma, Neuroscience, Neurology

## Abstract

Neuroscientific and clinical studies on auditory perception often use headphones to limit sound interference. In these conditions, sounds are perceived as internalized because they lack the sound-attributes that normally occur with a sound produced from a point in space around the listener. Without the spatial attention mechanisms that occur with localized sounds, auditory functional assessments could thus be underestimated. We hypothesize that adding virtually externalization and localization cues to sounds through headphones enhance sound discrimination in both healthy participants and patients with a disorder of consciousness (DOC). Hd-EEG was analyzed in 14 healthy participants and 18 patients while they listened to self-relevant and irrelevant stimuli in two forms: diotic (classic sound presentation with an “internalized” feeling) and convolved with a binaural room impulse response (to create an “externalized” feeling). Convolution enhanced the brains’ discriminative response as well as the processing of irrelevant sounds itself, in both healthy participants and DOC patients. For the healthy participants, these effects could be associated with enhanced activation of both the dorsal (where/how) and ventral (what) auditory streams, suggesting that spatial attributes support speech discrimination. Thus, virtually spatialized sounds might “call attention to the outside world” and improve the sensitivity of assessment of brain function in DOC patients.

## Introduction

Auditory signals in normal every-day life situations come from a source positioned in the space around the listener. Sound localization is possible due to slight binaural differences in time and level, as well as reverberation within the space surrounding the subject. Contrary to these normal listening situations, neuroscientific and clinical studies almost exclusively use diotic sounds (identical signals in both ears) and headphones to investigate auditory perception. This is especially true within clinical routines due to environmental noise. However, such listening creates the impression that the sound is located inside the head as it lacks the binaural cues of normal listening situations.

Auditory cortical processing of everyday situations is associated with an anatomo-functional dual stream, similar to the ones found in for example vision and language (for a review see^1^). The ventral pathway has been functionally conceptualized as the “what” stream, responsible for stimulus identification and recognition. The dorsal pathway constitutes the “where/how” stream, responsible for spatial processing, sensorimotor mapping, and guidance of action towards objects in space^2–5^. The dorsal stream can facilitate object recognition through the enabling of top-down attentional control processes within the ventral stream, which leads to a shift in attention to those object-features relevant for identification^6–9^. This means that listening to diotic sounds might not engage the spatial attention mechanisms and might not facilitate object recognition via the interaction between the dorsal and ventral streams. Thus, the evaluation of auditory recognition could be underestimated when headphones with diotic sounds are used.

Luckily, auditory spatial perception can be induced over headphones by applying interaural time and level differences. This creates an effect of lateralization and is associated with activation of the posterior temporal cortex, the posterior parietal cortex and the superior frontal sulcus (including the premotor cortex), i.e. the auditory dorsal stream^10,11^. Convolving the sound with a head related transfer function (HRTF), taking into account sound filtering by the head and pinnae (in an anechoic room), gives the impression of a sound-source outside the head^12^. The presentation of these “externalized” sounds is associated with enhanced activations of the auditory “where/how” pathway, among others in the posterior superior temporal gyrus and inferior parietal lobule^13^. They furthermore allow earlier brain responses, faster reaction times, and supplementary cortical processing^14^. A significantly stronger externalization percept can be obtained by convolving the sound with a binaural room impulse response (BRIR) that is a HRTF recorded in a room^15,16^. The included room-reverberation might give further indication of the properties of the space. The effects of such realistic spatialized sounds (i.e., convolution by a BRIR) on behavioral and cerebral processing has so far received surprisingly little attention.

Audition is the main modality used to assess cognition in patients in acute or prolonged disorder of consciousness (DOC) such as unresponsive wakefulness syndrome^17^, minimally conscious state^18^, or cognitive motor dissociation^19^. We hypothesis that the use of virtually externalized and localized auditory stimuli could increase top-down attention and thus discrimination to sounds, probably because of an interaction between the ventral and the dorsal auditory streams. Such effects should be present in healthy participants and patients with a DOC, but would be especially important in clinical settings where headphones are often necessary to limit surrounding noise. To test this hypothesis, we compared cerebral responses (using hd-EEG) to self-relevant (subject’s own name) and irrelevant stimuli (unfamiliar first names) presented in a diotic (classical sound presentation and perceived as “internalized”) or in a convolved form (using a BRIR to achieve an “externalized” percept).

## Materials and methods

### Participants

Data from 17 healthy participants was initially acquired. Three of these needed to be excluded due to excessive movement or eye-blinks. Data of 14 healthy participants could be analysed (mean age = 34.5, sd = 14.7 years; 8 female).

Eighteen patients with Disorders of Consciousness (DOC) were assessed at the Department of Intensive Care or at the Neurological Rehabilitation Service in the Neurological Hospital of Lyon (mean age = 47.5, sd = 19.4 years; 4 female). For 8 patients’ data was acquired less than 30 days after onset of coma. 7 had a traumatic etiology, 9 anoxic and 2 mixed causes (for demographics see table 1 in supplementary material). The patients were stable, non-sedated, and had no indication of deafness (i.e., brainstem auditory evoked potentials were present). The patients were in a state of coma (nr = 1), vegetative state/unresponsive wakefulness syndrome (nr = 7), or minimally conscious state (nr = 10) according to clinical consensus and coma recovery scale revised scores (CRS-R^20^). Behavioral assessments were performed on multiple occasions in the week(s) prior to the study protocol by clinical staff familiar with the CRS-R as part of clinical routine. One detailed CRS-R was performed by the researchers on the day of EEG assessment. This study was approved by the local Ethics Committee “CPP Sud-Est II” (2012-036-2), and all experiments were performed in agreement with the guidelines of the Declaration of Helsinki. Healthy participants or family representatives gave written informed consent. Healthy participants furthermore received monetary compensation for their time.

### Stimuli

We have adapted a well-documented protocol investigating the discrimination of self-relevant stimuli (the subject’s own name; SON) against irrelevant stimuli (other names; ON) in healthy participants and DOC patients^21,22^. Specifically, similar to^23^, these names were preceded by either preferred music or neutral control sounds (broadband noise). Presently, all stimuli were furthermore presented in either a diotic version (like previous studies) or in a convolved version.

All names were disyllabic (1.05 s, SD = 0.05 s). The SON was selected for each subject. Six irrelevant names were selected by asking participants or representatives to indicate on a predefined list of 16 names if any were familiar or not. All names were pronounced by three different voices, created using text to speech software (Two female, one male; Natural Reader, NaturalSoft Ltd.).

Six preferred music pieces were selected on the basis of a questionnaire filled in by participants or patients’ representative. This questionnaire assembled songs with high scores on affinity, familiarity and autobiographical memory within the French population^24^. Neutral sounds were similar for each subject and the same as in a previous study^25^. They consisted of a stationary noise with a spectral approximation of music, but did not share other acoustic characteristics (e.g., pitch, rhythm, envelope, or timbre). Each music or neutral sound stimuli had a duration of 30 s (± 2 s).

A convolved version of all stimuli (names, music, neutral sounds) was created with a BRIR. This BRIR was measured in a large cinema style room, with 418 soft seats and low ceilings where the loudspeaker was placed at 60 and 1 m away from the dummy head used for the recording^26^. A 60 azimuth was chosen to achieve maximum impressions of externalization^16^. To create externalized stimuli, the music, neutral sounds, and names were convolved with this BRIR, creating the impression that the sound originated within the large room. There were three versions of each stimulus: a non-convolved diotic version (with identical signals at both ears), a version externalized to the left, and a version externalized to the right. The latter was created simply by inversing the left and right ear signals obtained after convolution by the BRIR.

All stimuli were equalized to the same A-weighted sound level, and presented binaurally during the experiment at a sound pressure level of approximatively 65 dBA SPL. In patients, if environmental noise was high, the presentation level was slightly increased to a level that was clearly audible but not painful.

### Protocol

The whole protocol held 24 blocks of stimuli and the inter stimulus interval between two blocks was 1 s. One block was constituted by a 30 s excerpt of music (M) or neutral sound (N), followed by a sequence of names. Name sequences consisted of 42 names, each of the 6 ON and the SON repeated 6 times in a semi-randomized fashion (i.e., no more than 2 subsequent repetitions of a name and relatively equal distribution of names throughout the sequence) with a stimulus interval of 500–600 ms (in randomized steps of 10 ms). Blocks were presented by series of three (e.g., MMM–NNN–MMM–NNN). Furthermore, all stimuli (sound and names) within each series of three blocks were either presented in their diotic (d) or convolved (c) versions, and for the convolved version either from left (l) or right (r) (e.g., MdMdMd–NclNclNcr–McrMclMcr–NdNdNd). This means that if a music/sound was convolved on one side, the following names within the block were necessarily on that side as well. This was done in order to enhance context effects. All stimuli were delivered using either Presentation software (Neurobehavioral Systems, Inc., Berkeley, CA, https://www.neurobs.com, version 19.7) or via Python scripts. A pause was introduced in the middle of the protocol to allow participants to briefly move, or in case care was needed for patients. The complete protocol lasted around 35 min.

### Behavioural data

**Figure 1:**
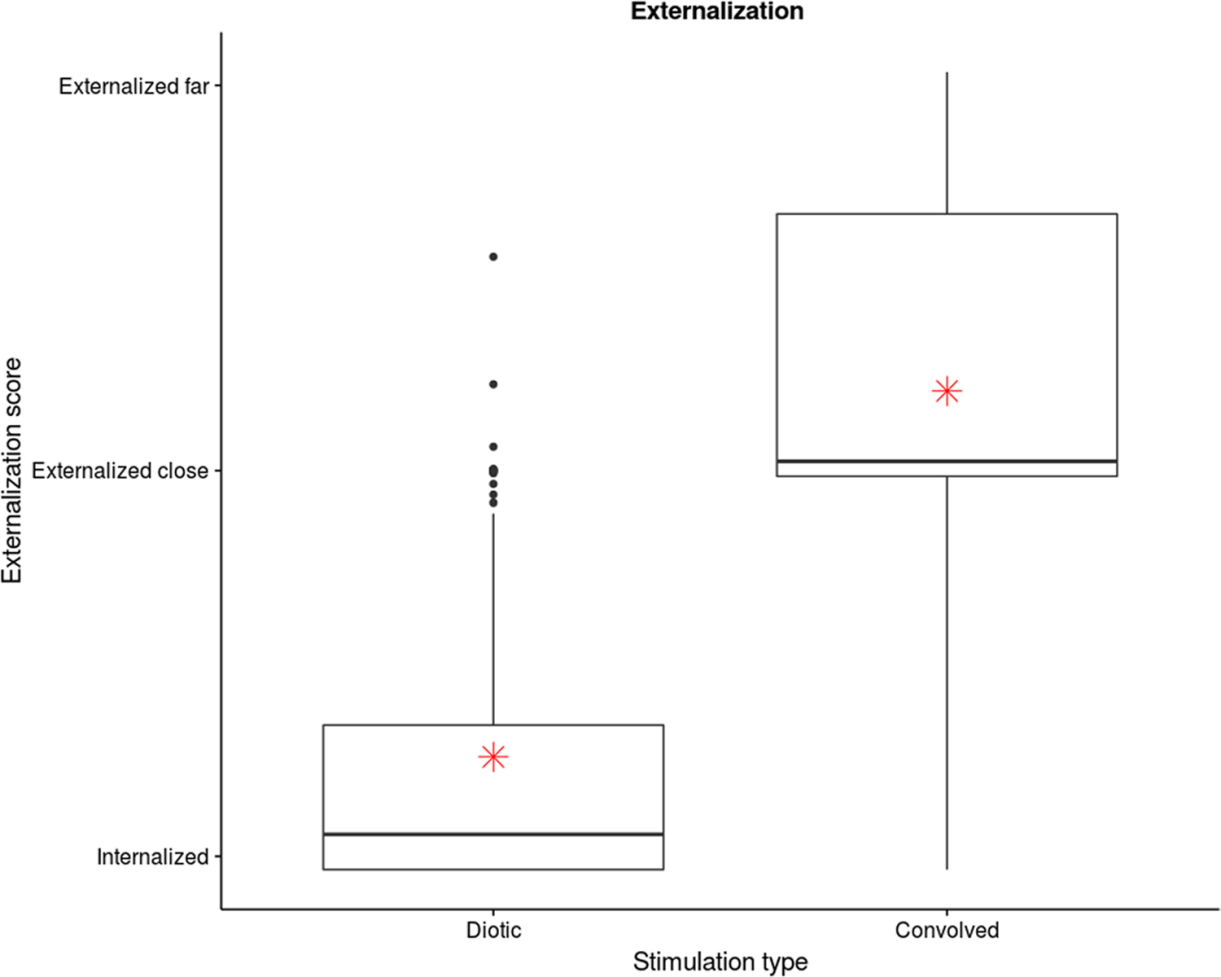
Behavioral evaluation of musical stimuli. Ratings for the subjective assessment of perceived externalization of all musical stimuli. Healthy participants listened again to all musical stimuli heard during the EEG protocol (both convolved and diotic versions), and rated using a slider on a continuous scale where the sound came from. Convolved sounds are generally judged as coming from the external environment, while diotic versions are judged as originating from within the head. Boxplot represents the confidence interval around the median. Star indicates the mean.

As a post-test, all healthy participants re-listened to all the music stimuli after the EEG paradigm in the same conditions as during EEG assessment. After each musical excerpt, participants were asked a series of questions. Using a continuous horizontal scale, they were asked how implicated they felt in the scene, how much emotions they felt, how familiar the excerpt was, how much they liked it, how much autobiographical memories they had, and how externalized the sound was. The scale for the last and most important question, was labelled as ‘in between the ears’ on one side of the scale, ‘outside the head but close’ in the middle of the scale, and ‘outside the head and far away’ on the other extremity. This post-test was administered using Presentation software (Neurobehavioral Systems, Inc., Berkeley, CA, https://www.neurobs.com, version 19.7) and ratings from the scale were used to assess differences between convolved and diotic music. Two-sided paired t-tests were performed using R, which showed that perceived externalization was significantly stronger for convolved songs (*p* = *2.2e−16, df* = *83*; Fig. 1). None of the other questions showed significant differences between diotic and convolved conditions (p > 0.05).

### EEG recording and analysis

Data was recorded using a sampling rate of 500 Hz with a 128-electrode geodesic sensor net (EGI^®^, philips) referenced to the vertex. Data was exported in its acquired format without any processing in EGI’s proprietary software. EEG analysis was done using MNE Python, version 15.1^27^. Data was first visually inspected to identify bad channels. Any channel with huge continuous outliers, or placed on open wounds, drains or bandages in the case of patients were indicated as bad. These were taken out of the analysis. Data was filtered between 0.1 and 40 Hz using a FIR zero-double filter and with a notch at 50 Hz. Patients’ analysis, without other alterations, was done with a 0.1 and 1 Hz highpass filter, and best results are represented. No consensus exists in the literature about highpass filters in brain damaged patients, and a 1 Hz filter was done to accommodate ERP analysis of those patients with overwhelming delta activity. Eye channels were recreated through subtraction of the channels above and below the eye, an average reference was taken and Cz was interpolated using the spherical spline method implemented in MNE. For any subject where data was affected by eye-blinks, an ICA was performed to remove the blink components from the signal. Data was epoched (from -200 to 1200 ms) and further analysis was performed on SON and the ON preceding it (for comparisons with similar signal-to-noise ratio). A baseline (− 200 to 0 ms) and a DC-detrend correction were performed. To further clean the data, an automatic rejection function was used^28^ (version of March 2018) where bad trials are either interpolated or rejected based on trial-wise assessment of individual sensor thresholds.

### Individual analyses

In each healthy participant and patient, epochs to SON and ON were compared in 4 conditions: when they were presented in their diotic (SONd vs ONd) or convolved versions (SONc vs ONc) and when they were presented after music (SONaM vs ONaM) or noise (SONaN vs ONaN). To test statistical differences between conditions, we used spatio-temporal clustering permutation tests^29^ with one sided t-tests and 20.000 permutations. Cluster level alpha was set to 0.01 with a cluster forming threshold of 0.05.

### Group analyses

For each healthy participant and patient, epochs were averaged according to names and/or conditions: SON and ON (all conditions confounded), SONd and ONd (diotic condition), SONc and ONc (convolved condition), SONaM and ONaM (music condition), SONaN and ONaN (noise condition). Group analysis first assessed the main effect of first names by comparing the differences between the individual ERPs to subject’s own name (SON) and the ERPs to the other names (ON), i.e. convolved/diotic or music/noise conditions confounded. Then, the main effect of convolution was assessed by comparing the differences between the ERPs to convolved first names and the ERPs to diotic first names: SONc vs. SONd, ONc vs. ONd, (SONc + ONc) vs. (SONd + ONd), and (SONc-ONc) vs. (SONd-ONd). The main effect of music was assessed by comparing the differences between the ERPs to first names following music and the ERPs to first names following noise: SONaM vs. SONaN, ONaM vs. ONaN, (SONaM + ONaM) vs. (SONaN + ONaN), and (SONaM-ONaM) vs. (SONaN-ONaN).

For healthy participants, these analyses were performed using spatio-temporal clustering permutation tests^29^ using one sided t-tests and 20.000 permutations. Cluster level alpha was set to 0.01 with a cluster forming threshold of 0.05. For the patient population, these analyses were performed within the unartefacted electrodes over all patients (N = 40), as interpolation was not suitable for most of them. Due to the loss of spatial covering within the grand average of patient data, because of the deleted channels, the group analysis consisted of temporal clustering with one sided t-tests and 20.000 permutations within each electrode. Again, an alpha of 0.01 and a cluster forming threshold of 0.05 was used.

### Source reconstruction

**Figure 2:**
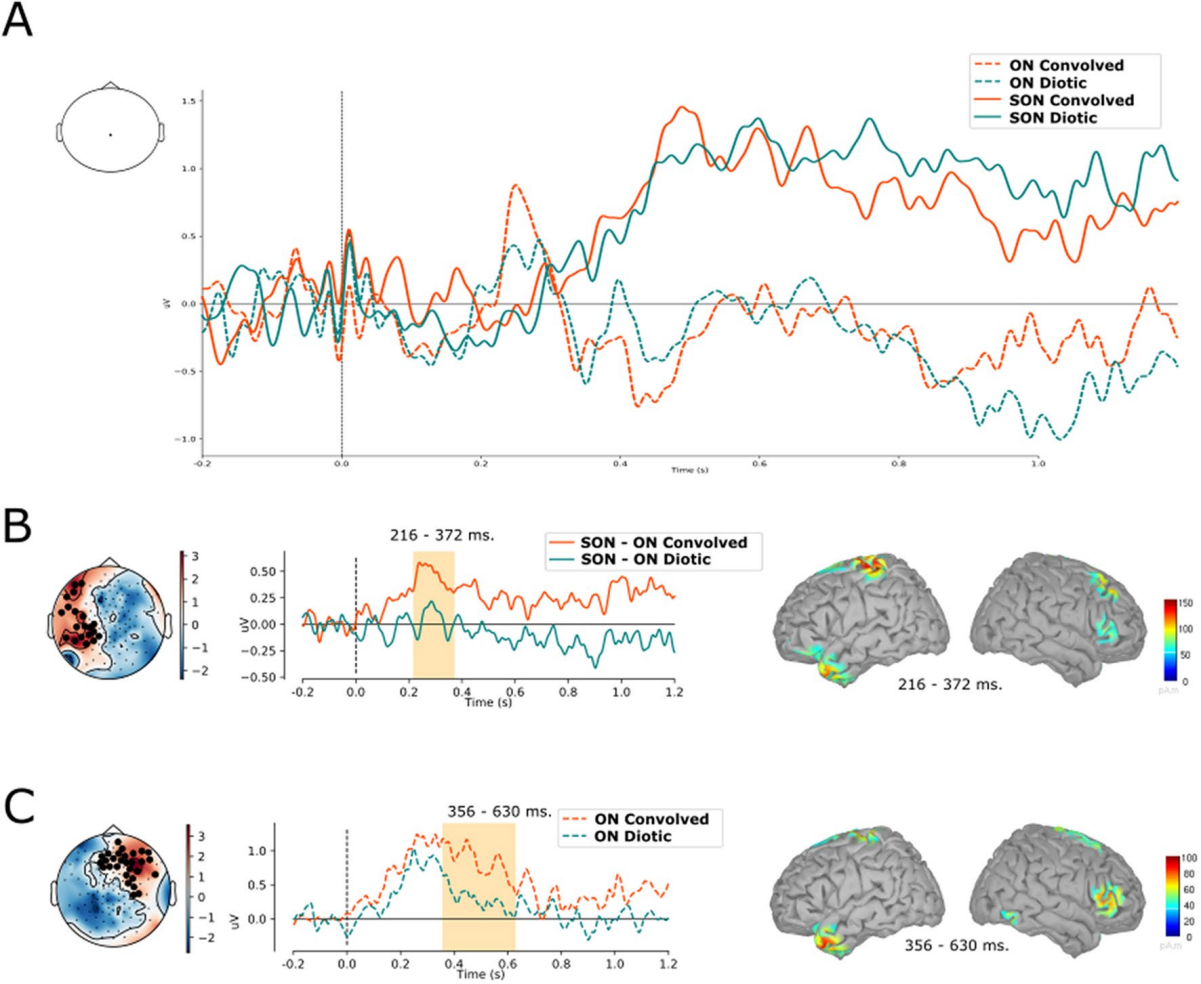
Comparison of evoked responses. **(A)** Comparison of evoked responses within the group of healthy subjects in convolved (red) and diotic (teal) conditions after self-relevant (solid lines: subjects own name) or irrelevant stimuli (dotted lines; other names). **(B)** Comparison of differences between own and other names between convolved and diotic conditions (SONc-ONc vs SONd-ONd). **(C)** Comparison of the other name between convolved and diotic conditions (ONc vs ONd). Significant clusters after spatiotemporal cluster permutation testing are indicated by black electrodes on each averaged map (within the timeframe of significance). ERP traces are averaged activity within significant clusters of electrodes, and significant timeframe is indicated with orange. Source localisation represents these differences within the timeframe of this cluster.

The cortical sources of the significant differences observed in the healthy group’s event related potential (ERP) analyses were reconstructed using the Brainstorm software^30^. We used the MNI anatomical template “Colin 27”, the openMEEG BEM model, the adjoint formulation and the identity matrix as noise covariance. Source reconstruction was done on the significant temporal difference between two conditions in each individual and the individual source reconstructions were averaged. The minimum norm imaging method was used with the current density map measure and constraint dipole orientations. For cortical surface visualization, we used a color bar maximum of 150 pA.m or 100 pA.m (Fig. 2B,C, respectively), with a threshold of 35% and a minimum size of 10.

### Data availability

Due to the clinical nature of patient data and institutional restriction, data are available from the corresponding author upon request.

## Results

Within healthy participants, the group analysis of ERPs showed a first name effect that is illustrated by a significant difference between the subject’s own first name (SON) and the other first names (ON) from 410 ms until the end of the epoch, associated to the classical P3 effect. This significant effect can be observed in both the convolved and diotic conditions. Figure 2A shows the average evoked potentials for the SON and ON in both the convolved and diotic conditions on one single centrally located electrode.

### Effect of convolution on name discrimination in healthy participants

A main effect of convolution was observed and was illustrated by a significant difference, from 216 to 372 ms (associated to the N2 wave), between (SONc-ONc) and (SONd-ONd), on a cluster of left parietal electrodes. Reconstructing the difference showed a source within the left primary somatosensory area and left primary motor region, extending into the superior frontal region, as well as in the left temporal pole, and right pars triangularis and right premotor regions (Fig. 2B).

When directly comparing SON between convolved and diotic (SONc vs SONd), no difference was observed. In contrast, the comparison between convolved and diotic other names (ONc vs ONd) showed a significant difference, between 356 and 630 ms. Source reconstruction within this time-window indicated that these differences could be located in the right pars triangularis found within the inferior frontal area, in the left temporal pole and in the bilateral pre and post central gyrus (Fig. 2C).

Individual statistics on epochs showed differences for both conditions in 6/14 healthy participants for the SONd vs ONd comparison and in 9/14 healthy participants for the SONc vs ONc comparison (thus, we observed a relative increase of 50% in significant differences between SON and ON within the convolved condition).

### Effect of convolution on name discrimination in patients with DOC

**Figure 3:**
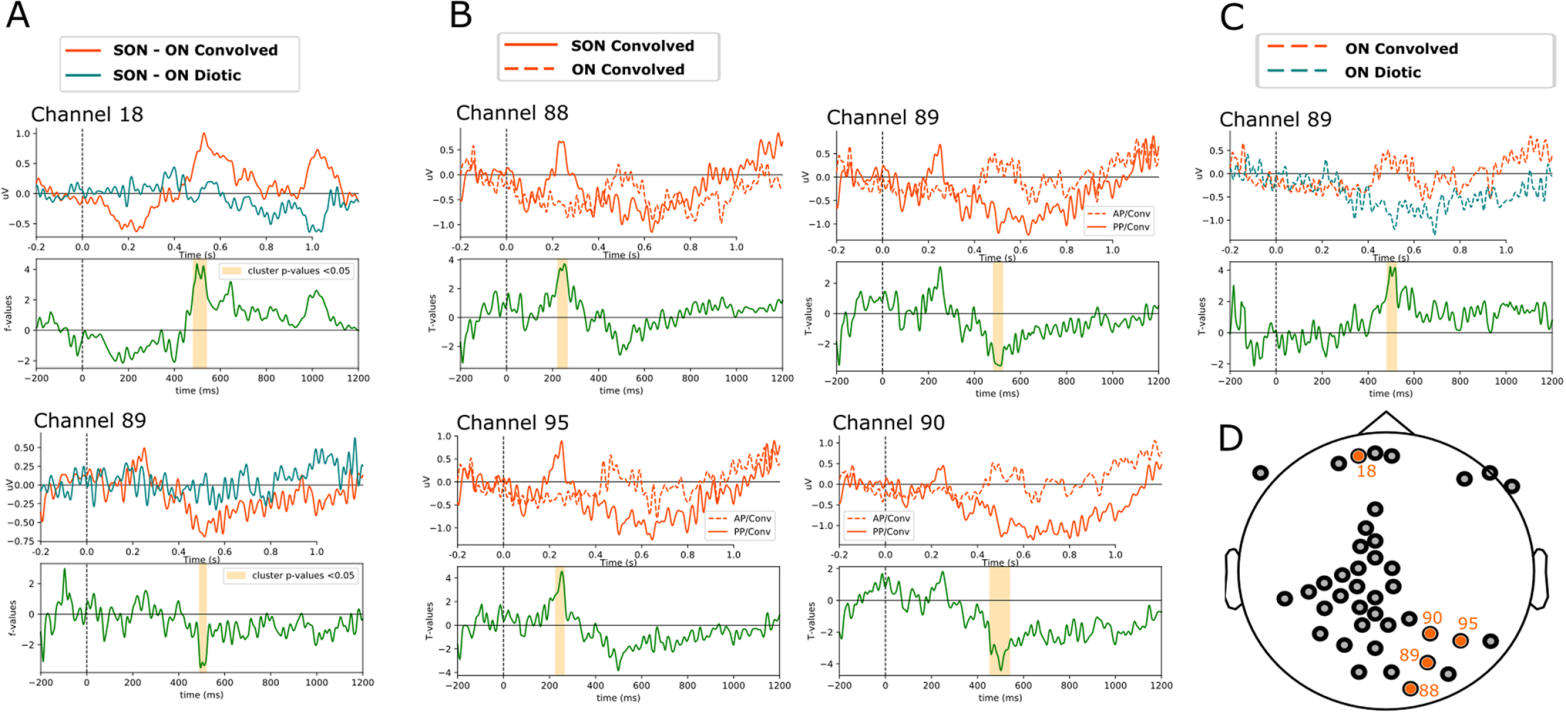
Comparison of evoked responses in patients. Comparison of evoked responses within patients in convolved (red) and diotic (teal) conditions for the own name (solid) and other names (dotted). Each channel figure consists of the comparison between indicated conditions (top) and the t-values of the difference in green (bottom) with significant cluster indicated in yellow. (**A)** Comparison of differences between own and other names between convolved and diotic conditions (SONc-ONc vs SONd-ONd). **(B)** Comparison between the own and other names within the convolved condition (SONc vs ONc). **(C)** Comparison of the other name between convolved and diotic conditions (ONc vs ONd). **(D)** Topography of assessed electrodes. Location of significant electrodes mentioned in the figure are indicated in orange.

Due to the low sample of electrodes that are good for all patients and the large heterogeneity of patients’ clinical profiles, results of patient group analyses need to be interpreted with great caution. These analyses did not show the global effect of first names (SON vs ON), but a significant difference in SON and ON between the convolved and the diotic condition (SONc-ONc vs SONd-ONd) was found from 482 to 540 ms (i.e. in the P3 wave window) within two electrodes (Fig. 3A). Furthermore, only within the convolved condition itself, a difference between SON and ON could be observed (SONc vs. ONc) within four electrodes and around the classical N2-P3 timeframes (Fig. 3B). No difference was observed when directly comparing SON between convolved and diotic (SONc vs SONd), but a significant difference was observed between 482 and 524 ms when comparing ON between convolved and diotic (ONc vs ONd; Fig. 3C).

Individual analyses of patients showed 5/18 patients with cluster(s) of significant difference between SON and ON in the convolved condition (SONc vs ONc). Only 2/18 patients showed a difference between SON and ON in the diotic condition (SONd vs ONd), one of which also showed a difference in the convolved condition (supplementary Fig. 1). Of the 5/18 patients showing a significant difference in the convolved condition, 3/5 showed improvements in subsequent states, and 1/5 patient remained stable (our assessment was over 6 years after onset). The patient showing a discriminating response in both conditions (convolved and diotic) died not long after due to medical issues unrelated to, but influencing cognitive state.

### Effect of music on SON discrimination

As previously reported^23^, no effect of music was observed within the group analyses of healthy participants: for (SONm-ONm) vs (SONn-ONn), SONm vs SONn and ONm vs ONn.

In DOC patients, and as previously reported^23^, an effect of music was observed in the group analysis (see supplementary figures). It was illustrated by a difference between music and noise from 374 to 404 ms within one electrode for SONm vs SONn, and from 710 to 840 ms within two electrodes for (SONm + ONm) vs (SONn + ONn). A difference between SON and ON within the music condition was also observed within three electrodes from 92 to 278 ms.

Individual statistics in patients showed 6/18 patients with a significant difference between SON and ON following music (SONaM vs ONaM) and 2/18 within the noise condition (SONaN vs ONaN). One of these had previously shown differences in both convolved and diotic conditions (supplementary Fig. 2). No interaction between music and convolution could be performed due to the low signal to noise ratio (i.e., not enough epochs in each condition alone).

## Discussion

In the present study, we assessed the effect of realistic spatialized sounds on speech discrimination, as compared to diotic sounds (which is the classical way of presenting sounds with headphones) in both healthy conscious participants and patients with a disorder of consciousness (DOC). To this aim, we compared the EEG brain responses to convolved sounds (with a BRIR at 60 degree azimuth), to the brain responses to diotic sounds, which are perceived as internalized. In line with previous research, the convolved sounds were associated with an enhanced feeling of externalization^16^. Within both healthy participants and patients, realistic spatialized sounds enhanced discrimination between self-relevant (subjects own name; SON) and irrelevant (other name; ON) sounds, as well as the processing of irrelevant sounds itself.

### Healthy participants

In healthy participants, the convolution seemed to cause a stronger discrimination of self-relevant as compared to irrelevant sounds (SON-ONc vs SON-ONd; Fig. 2B) and a stronger detection of the irrelevant sounds (ONc vs ONd; Fig. 2C). These two effects seem to be associated with an extended activation of the auditory dorsal and ventral pathways. Although source reconstruction should be viewed with extreme vigilance due to, among others, the lack of individual MRI acquisition and a small group, the dataset showed within the healthy participants that both effects were associated with the activation of the somatosensory and premotor regions, as well as of the pars triangularis and the left temporal pole (Fig. 2B).

In a previous fMRI study, convolved sounds with individual HRTFs enhanced activity in the bilateral posterior temporal gyri and in the superior and inferior parietal lobules^13^. The present study suggests that the somatosensory and premotor regions could also participate in sound perception when the acoustical properties of the sounds are associated with a stronger feeling of externalization. Interestingly, these structures are part of the dorsal pathway, which connects sensory areas with posterior/inferior parietal and prefrontal regions and which is responsible for spatial processing (including location, relative position, and motion), sensorimotor mapping and guidance of action towards objects in space^1^. This suggests that externalized sounds are probably responsible for an extended activation of the body space representation, i.e. including both the person who perceives and the sound in space. This is in accordance with studies showing that peripersonal space representations (space representations centered on different body parts) arise through extensive multisensory interactions within a set of interconnected parietal and frontal regions^31^, like the premotor cortex^32^.

The present study also suggests that the processing of convolved sounds could be associated with an extended activation of the pars triangularis and the left temporal pole. These structures are part of the auditory ventral pathway, which connects the ventral temporal structures to the ventral prefrontal regions and which is responsible for stimulus identification and recognition, the mapping of information onto conceptual representations, and the comprehension of spoken (and written) language^1^. In particular, the activation of the pars triangularis seems to be associated with linguistic processes, including semantics^33^, and has been proposed to be crucial in a language-control network^34^. The left temporal pole on the other hand can be seen as a region combining semantic and conceptual knowledge^35^.

An extended activation of the ventral stream in the present study can seem surprising at first, as stimuli with the same linguistic contents were contrasted. We rather suggest that an extended activation of the “what” pathway was a consequence of an extended activation of the “where/how” pathway, because of the body space properties of convolved sounds. This is in accordance with the hypothesis that the dorsal stream, which is associated with a fronto-parietal control network, could facilitate object recognition through the enabling of top-down attentional control processes within the ventral stream^6–9^. Thus, convolved sounds would lead to a shift in attention to their relevant properties for identification.

To sum, the present study suggests that virtually spatialized sounds were processed as if they were coming from outside of the body, allowing for an improved top-down attentional processing and thus for an extended lexical processing. This would be true for SON-ON discrimination but also for ON processing itself, suggesting an overall attentional rather than a local attentional effect on relevant stimuli. Another possibility is that the information is simply available to a wider network in the brain and therefore more easily detectable when simulated outside of the head.

### Patients with DOC

Realistic spatialized sounds might also enhance auditory processing in DOC patients. Statistical analysis for the patient population is unfavorably limited by clinical reality concerning sample size and heterogeneity, and the data suffers from a multiple comparison issue across electrodes on the group level. Results are thus exploratory and should be interpreted with caution. With this in mind, an effect of convolution on SON-ON discrimination (SON-ONc vs SON-ONd) could be observed but occurred later as compared to healthy participants (around 500 ms and 300 ms, respectively). Even if the two populations differed in age, impairment in cognitive function and delays in speech perception have been previously reported^21,22,36^. The second effect of convolution on irrelevant sounds could also be observed (ONc vs ONd; Fig. 3C), but with a similar latency (around 500 ms) compared to healthy participants.

Convolved sounds might improve speech discrimination in DOC patients at a group level but also at an individual level. Indeed, individual statistical differences between SON and ON could be observed more often within the convolved condition than within the diotic condition (in 5 and 2 patients, respectively). The enhanced probability to observe differences during the convolved condition might mean improved sensitivity of the test, which could translate to a more accurate evaluation of residual cognitive processing. This is in line with the fact that the SON-ON difference can only be observed in the convolved condition (and not in the diotic condition) in the group analysis, whereas it could be obtained in both convolved and diotic conditions in the group analysis of healthy subjects.

Increased brain responses after salient stimuli have been observed in the literature for DOC patients. Especially within the auditory system, sensitivity of observations^25,37,38^, as well as brain function^39–41^ can be improved through salient stimuli. In fact, integrity of the auditory system might be especially important for diagnostic assessment^42^, and automatic classification efforts seem to point towards highest discriminatory possibilities in the auditory network^43^. Our results confirm previous findings of a ‘boosting’ effect of music on stimuli discrimination^23^, and show that a simple adaptation of the sound itself to create an externalized impression might also ‘boost’ such discrimination. However, due to protocol length limitations we are currently unable to assess an interaction (music by convolution), and are unable to distinguish if these two effects are additive or if a patients’ maximum capacity of brain functioning can be obtained with either. Furthermore, the present study does not allow us to reliably compare in healthy participants nor patients, the different voices used for the name sequences or the side of auditory stimulation. However, different voices were used for variation in the protocol and the randomisation of all stimuli should have distributed any possible effect over conditions. Future studies could explore potential effects of voice or direction on ERP.

The use of realistic spatialized sounds might have participated in an enhanced body space perception in DOC patients. Two recent papers have shown that such residual peripersonal space representation can indeed be present in patients^44,45^. Both studies show, from different angles, the relation between the presence of peripersonal space processing and the level of consciousness and cognitive processing. As mentioned, a first-person perspective, meaning that the body is different from the environment, is necessary for self-consciousness^46,47^. Furthermore, it has been suggested that bodily self-consciousness is a necessary requirement for consciousness^48^. Thus, by allowing that the sound does not originate in ones’ head, the convolved sounds might have improved the patients’ residual awareness.

We could also hypothesize that the improved spatial attributes of the convolved sounds might facilitate auditory processing in patients through, as in healthy participants, the interaction of the dorsal and ventral pathways, and thus makes discrimination easier in the convolved condition. If the convolution improves top-down attentional control processes within the ventral stream, it might be responsible for a shift in attention to relevant (SON), as well as to irrelevant (ON) sounds. Indeed, an effect of convolution could also be observed for ON detection, at a similar latency than in healthy participants. Thus, one may suggest that the processing of irrelevant stimuli was more similar to that observed for healthy participants because it might be associated with an automatic detection, unlike what happens for the processing of relevant stimuli, which could be associated with awareness, thus with additional neural resources.

In sum, our results indicate that when using headphones, virtually externalized and localized sounds created through convolution with a BRIR have effects on sound discrimination, in healthy conscious participants and possibly in DOC patients as well. Such a convolution enhances reality through the impression that an auditory signal is coming from a specific point in the space around the listener, as if the subject is “called to the outside world”. Our data indicate that this might improve sound discrimination and auditory awareness in general. This might be an important finding, especially in those cases where improved sensitivity of assessments can have a strong impact, such as in those patients where a dissociation between behavioral responsiveness and cognitive functioning is possible (i.e., disorders of consciousness). We thus advocate a systematic convolution of the auditory signal.

## Supplementary Information

Supplementary Information.

## References

[1] Cloutman LL (2013). Interaction between dorsal and ventral processing streams: Where, when and how?. Brain Lang..

[2] Ahveninen, J., Kopčo, N. & Jääskeläinen, L. Psychophysics and neuronal bases of sound localization in humans. *Hear. Res.* **207** (2014). 10.1016/j.heares.2013.07.008 PMC3858499 23886698

[3] Kopčo N (2012). Neuronal representations of distance in human auditory cortex. Proc. Natl. Acad. Sci..

[4] Formisano E (2003). Mirror-symmetric tonotopic maps in human primary auditory cortex. Neuron.

[5] Zündorf, I. C., Lewald, J. & Karnath, H.-O. Neural correlates of sound localization in complex acoustic environments. *PLOS ONE* **8**, e64259 (2013). 10.1371/journal.pone.0064259 PMC3653868 23691185

[6] Jaaskelainen IP (2004). Human posterior auditory cortex gates novel sounds to consciousness. Proc. Natl. Acad. Sci..

[7] Laycock R, Crewther DP, Fitzgerald PB, Crewther SG (2009). TMS disruption of V5/MT+ indicates a role for the dorsal stream in word recognition. Exp. Brain Res..

[8] Wang W-J, Wu X-H, Li L (2008). The dual-pathway model of auditory signal processing. Neurosci. Bull..

[9] Martínez A (1999). Involvement of striate and extrastriate visual cortical areas in spatial attention. Nat. Neurosci..

[10] Kong L (2014). Auditory spatial attention representations in the human cerebral cortex. Cereb. Cortex.

[11] Alain C, Arnott SR, Hevenor S, Graham S, Grady CL (2001). “What” and “where” in the human auditory system. Proc. Natl. Acad. Sci..

[12] Wightman, F. L. & Kistler, D. J. Headphone simulation of free‐field listening. II: Psychophysical validation. *J. Acoust. Soc. Am.* **85**, 868–878 (1989). 10.1121/1.397558 2926001

[13] Callan A, Callan DE, Ando H (2013). Neural correlates of sound externalization. Neuroimage.

[14] Getzmann S, Lewald J (2010). Effects of natural versus artificial spatial cues on electrophysiological correlates of auditory motion. Hear. Res..

[15] Catic J, Santurette S, Dau T (2015). The role of reverberation-related binaural cues in the externalization of speech. J. Acoust. Soc. Am..

[16] Leclère T, Lavandier M, Perrin F (2019). On the externalization of sound sources with headphones without reference to a real source. J. Acoust. Soc. Am..

[17] Laureys S (2010). Unresponsive wakefulness syndrome: A new name for the vegetative state or apallic syndrome. BMC Med..

[18] Bruno MA (2012). Functional neuroanatomy underlying the clinical subcategorization of minimally conscious state patients. J. Neurol..

[19] Owen AM (2020). Improving diagnosis and prognosis in disorders of consciousness. Brain.

[20] Giacino JT, Kalmar K, Whyte J (2004). The JFK Coma Recovery Scale-revised: Measurement characteristics and diagnostic utility11No commercial party having a direct financial interest in the results of the research supporting this article has or will confer a benefit upon the authors or upon any organization with which the authors are associated. Arch. Phys. Med. Rehabil..

[21] Perrin F (2006). Brain response to one’s own name in vegetative state, minimally conscious state, and locked-in syndrome. Arch. Neurol..

[22] Schnakers, C. *et al.* Voluntary brain processing in disorders of consciousness. **7** (2008). 10.1212/01.wnl.0000334754.15330.69 19001251

[23] Castro M (2015). Boosting cognition with music in patients with disorders of consciousness. Neurorehabil. Neural Repair.

[24] Jakubowski, K., Eerola, T., Tillmann, B., Perrin, F. & Heine, L. A cross-sectional study of reminisence bumps for music-related memories in adulthood. *Music Sci.* **3**, 1–13 (2020).

[25] Heine L (2017). Effects of preference and sensory modality on behavioural reaction in patients with disorders of consciousness. Brain Inj..

[26] Hummersone, C., Mason, R. & Brookes. Dynamic precedence effect modeling for source separation in reverberant environments. *IEEE Trans. Audit. Speech Lang. Process.* **18**, 1867–1871.

[27] Gramfort A (2014). MNE software for processing MEG and EEG data. Neuroimage.

[28] Jas, M. *et al. MEG/EEG Group Study with MNE: Recommendations, Quality Assessments and Best Practices*. 10.1101/240044 (2017).

[29] Maris E, Oostenveld R (2007). Nonparametric statistical testing of EEG- and MEG-data. J. Neurosci. Methods.

[30] Tadel F, Baillet S, Mosher JC, Pantazis D, Leahy RM (2011). Brainstorm: A user-friendly application for MEG/EEG analysis. Comput. Intell. Neurosci..

[31] di Pellegrino G, Làdavas E (2015). Peripersonal space in the brain. Neuropsychologia.

[32] Graziano MSA, Reiss LAJ, Gross CG (1999). A neuronal representation of the location of nearby sounds. Nature.

[33] Friederici AD, Gierhan SME (2013). The language network. Curr. Opin. Neurobiol..

[34] Elmer, S. Broca pars triangularis constitutes a “hub” of the language-control network during simultaneous language translation. *Front. Hum. Neurosci.* **10** (2016). 10.3389/fnhum.2016.00491 PMC5040713 27746729

[35] Schneider B, Heskje J, Bruss J, Tranel D, Belfi AM (2018). The left temporal pole is a convergence region mediating the relation between names and semantic knowledge for unique entities: Further evidence from a “recognition-from-name” study in neurological patients. Cortex.

[36] Risetti, M. *et al.* On ERPs detection in disorders of consciousness rehabilitation. *Front. Hum. Neurosci.* **7** (2013). 10.3389/fnhum.2013.00775 PMC3834290 24312041

[37] Cheng, L. *et al.* Do sensory stimulation programs have an impact on consciousness recovery? *Front. Neurol.* **9** (2018). 10.3389/fneur.2018.00826 PMC6176776 30333789

[38] Magee WL (2018). Music in the diagnosis, treatment and prognosis of people with prolonged disorders of consciousness. Neuropsychol. Rehabil..

[39] O’Kelly J (2013). Neurophysiological and behavioral responses to music therapy in vegetative and minimally conscious states. Front. Hum. Neurosci..

[40] Okumura Y (2014). Brain activation by music in patients in a vegetative or minimally conscious state following diffuse brain injury. Brain Inj..

[41] Carrière M (2020). An echo of consciousness: Brain function during preferred music. Brain Connect..

[42] Binder M, Górska U, Griskova-Bulanova I (2017). 40Hz auditory steady-state responses in patients with disorders of consciousness: Correlation between phase-locking index and Coma Recovery Scale-revised score. Clin. Neurophysiol..

[43] Demertzi A (2015). Intrinsic functional connectivity differentiates minimally conscious from unresponsive patients. Brain.

[44] Noel, J.-P. *et al.* Peri-personal space encoding in patients with disorders of consciousness and cognitive-motor dissociation. *NeuroImage Clin.* **24**, 101940 (2019). 10.1016/j.nicl.2019.101940 PMC6664240 31357147

[45] Calabrò RS (2020). Peri-personal space tracing by hand-blink reflex modulation in patients with chronic disorders of consciousness. Sci. Rep..

[46] Gallagher, S. & Zahavi, D. *Phenomenological Approaches to Self-Consciousness*. (2005).

[47] Serino A (2013). Bodily ownership and self-location: Components of bodily self-consciousness. Conscious. Cogn..

[48] Blanke O, Slater M, Serino A (2015). Behavioral, neural, and computational principles of bodily self-consciousness. Neuron.

