# Supplementary material for: Virtually spatialized sounds enhance auditory processing in healthy participants and patients with a disorder of consciousness
Source: Sci Rep. 2021 Jul 1;11:13702. doi: 10.1038/s41598-021-93151-6 (PMC8249625; doi:10.1038/s41598-021-93151-6)
Supplement: Supplementary file 1 — Supplementary Information. [file 41598_2021_93151_MOESM1_ESM.docx]

**Virtually spatialized sounds enhance auditory awareness in patients with a disorder of consciousness.**

**Supplementary material**

Lizette Heine^1,2^, Alexandra Corneyllie^1^, Florent Gobert^1,4^, Jacques Luauté^3,4^, Mathieu Lavandier^2^, Fabien Perrin^1*^

1: Audition Cognition and Psychoacoustics Team, Lyon Neuroscience Research Center, INSERM U1028, CNRS UMR5292, Lyon France.

2: Univ. Lyon, ENTPE, Laboratoire de Tribologie et Dynamique des Systèmes UMR 5513, Rue Maurice Audin, F-69518 Vaulx-en-Velin Cedex, France

3: Service de médecine physique et de réadaptation, rééducation neurologique, hôpital Henry-Gabrielle, CHU de Lyon, 69230 Saint-Genis-Laval, France

4: Trajectoires Team, Lyon Neuroscience Research Center, INSERM U1028, CNRS UMR5292, Lyon France.

**Corresponding author:**

Fabien Perrin

Audition Cognition and Psychoacoustics Team,

Lyon Neuroscience Research Center (UCBL, INSERM U1028, CNRS UMR5292)

Centre hospitalier le Vinatier – Bâtiment 462 – Neurocampus Michel Jouvet

95 Boulevard Pinel

69675 Bron cedex

Fabien.perrin@univ-lyon1.fr

**Table 1.**

| **Patient** | **Gender** | **Age** | **Days since onset** | **CRS-R** | | | | | | **CRS-R total** | **Etiology** | **CRS-R**  **Diagnosis** | **Outcome** |
| --- | --- | --- | --- | --- | --- | --- | --- | --- | --- | --- | --- | --- | --- |
|  |  |  |  | **Auditory** | **Visual** | **Motor** | **Oromotor** | **Communication** | **Attention** |  |  |  |  |
| 1 | F | 25 | 94 | 0 | 0 | 1 | 1 | 0 | 2 | 4 | Trauma | UWS | ~ |
| 2 | M | 36 | 23 | 0 | 0 | 5 | 1 | 0 | 1 | 7 | Trauma & Anoxic | MCS- | + |
| 3 | F | 65 | 2116 | 1 | 3 | 2 | 1 | 0 | 1 | 8 | Anoxic | MCS- | ~ |
| 4 | M | 28 | 76 | 3 | 5 | 4 | 1 | 0 | 1 | 14 | Trauma | MCS+ | + |
| 5 | M | 59 | 59 | 1 | 1 | 0 | 1 | 0 | 2 | 5 | Trauma | UWS | + |
| 6 | F | 49 | 48 | 1 | 3 | 2 | 1 | 0 | 2 | 9 | Trauma | MCS- | + |
| 7 | F | 74 | 41 | 1 | 0 | 0 | 0 | 0 | 2 | 3 | Anoxic | UWS | -- |
| 8 | M | 20 | 18 | 0 | 0 | 1 | 1 | 0 | 0 | 2 | Trauma & Anoxic | COMA | + |
| 9 | M | 61 | 30 | 1 | 3 | 2 | 1 | 0 | 1 | 8 | Anoxic | MCS- | + |
| 10 | M | 76 | 60 | 2 | 3 | 0 | 1 | 0 | 2 | 8 | Anoxic | MCS- | + |
| 11 | M | 52 | 36 | 3 | 3 | 2 | 0 | 0 | 2 | 10 | Anoxic | MCS+ | ~ |
| 12 | M | 63 | 18 | 0 | 0 | 2 | 1 | 0 | 1 | 4 | Anoxic | UWS | + |
| 13 | M | 70 | 14 | 0 | 0 | 0 | 1 | 0 | 1 | 2 | Anoxic | UWS | + |
| 14 | M | 47 | 10 | 0 | 0 | 2 | 1 | 0 | 1 | 4 | Trauma | UWS | ~ |
| 15 | M | 38 | 15 | NA on day of testing | | | | | | | Anoxic | UWS | -- |
| 16 | M | 21 | 68 | 3 | 0 | 5 | 0 | 0 | 1 | 9 | Trauma | MCS- | + |
| 17 | M | 18 | 11 | 0 | 1 | 3 | 1 | 0 | 1 | 6 | Trauma | MCS- | + |
| 18 | M | 53 | 16 | 0 | 0 | 2 | 1 | 0 | 2 | 5 | Anoxic | MCS- | -- |

**Patients’ demographics**

F=female, M=male, UWS=unresponsive wakefulness syndrome, MCS=minimally conscious state, ~ = no change in diagnosis at 6 months, + improved diagnosis at 6 months, -- = deceased.

**Supplementary figure 1.**

**
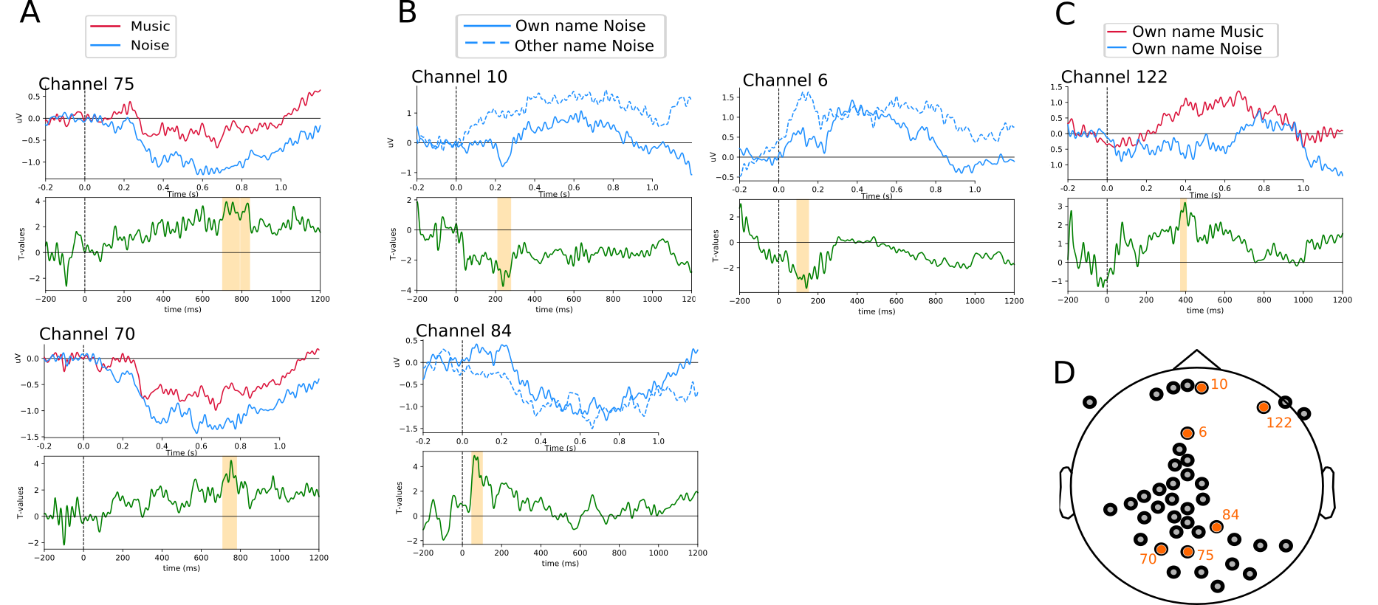
**

**Comparison of evoked responses for music in patients**

Comparison of evoked responses within patients in Music (red) and Noise (blue) conditions for the own name (solid) and other names (dotted). Each channel figure consists of the comparison between indicated conditions (top) and the t-values of the difference in green (bottom) with significant cluster indicated in yellow. **A)** Comparison of differences between music and noise. B) Comparison between the own and other names within the Noise condition. C) Comparison of the own name between music and noise conditions. D) topography of assessed electrodes. Location of significant electrodes mentioned in the figure are indicated in orange.

**Supplementary figure 2.**

**
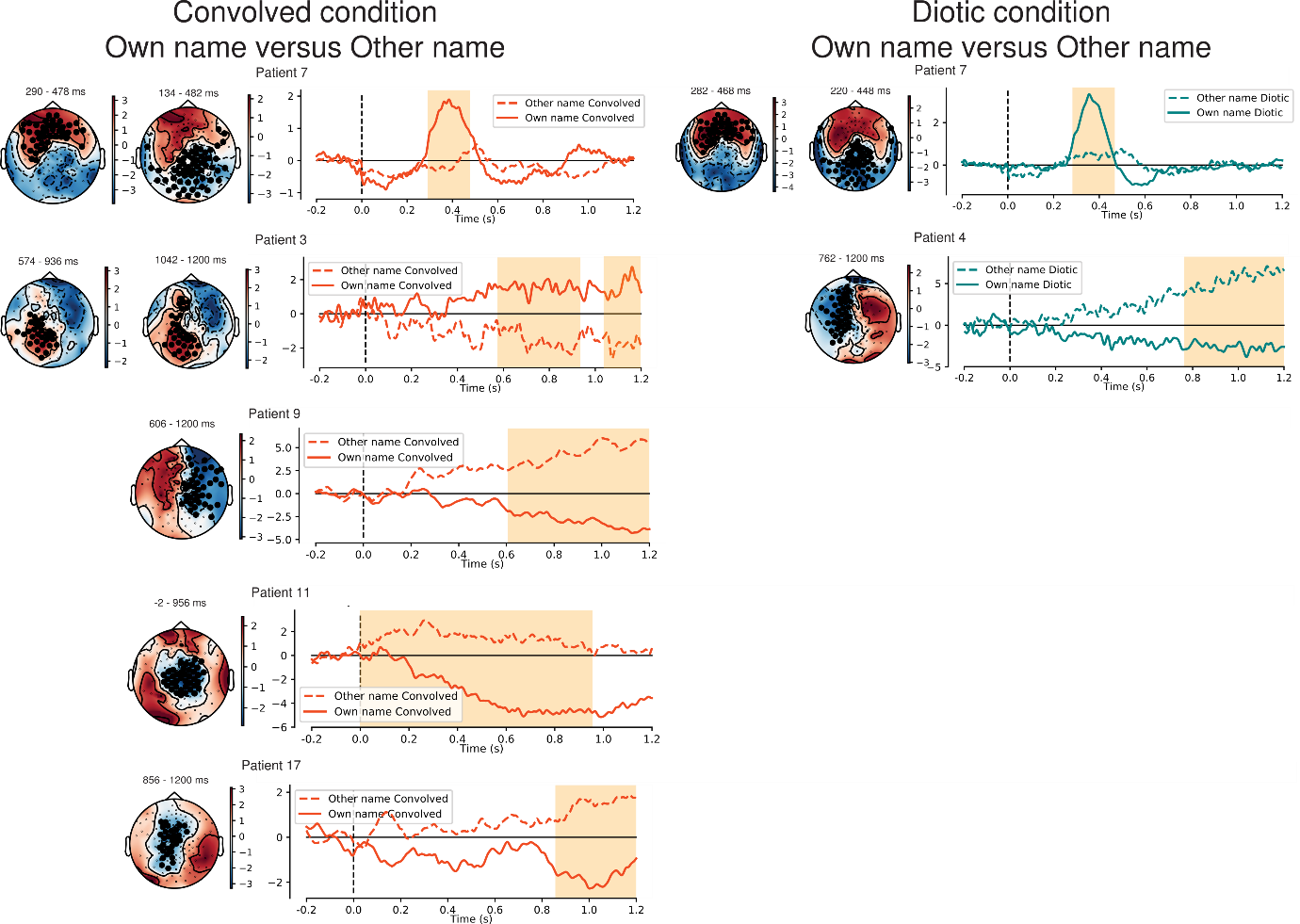
**

**Patients’ individual results convolved and diotic conditions.**

Results of spatiotemporal cluster permutation tests within the patients. Significant clusters are indicated by black electrodes on each averaged map (within the timeframe of significance). ERP traces are averaged activity within significant clusters of electrodes, and significant timeframe is indicated with orange.

**Supplementary figure 3.**


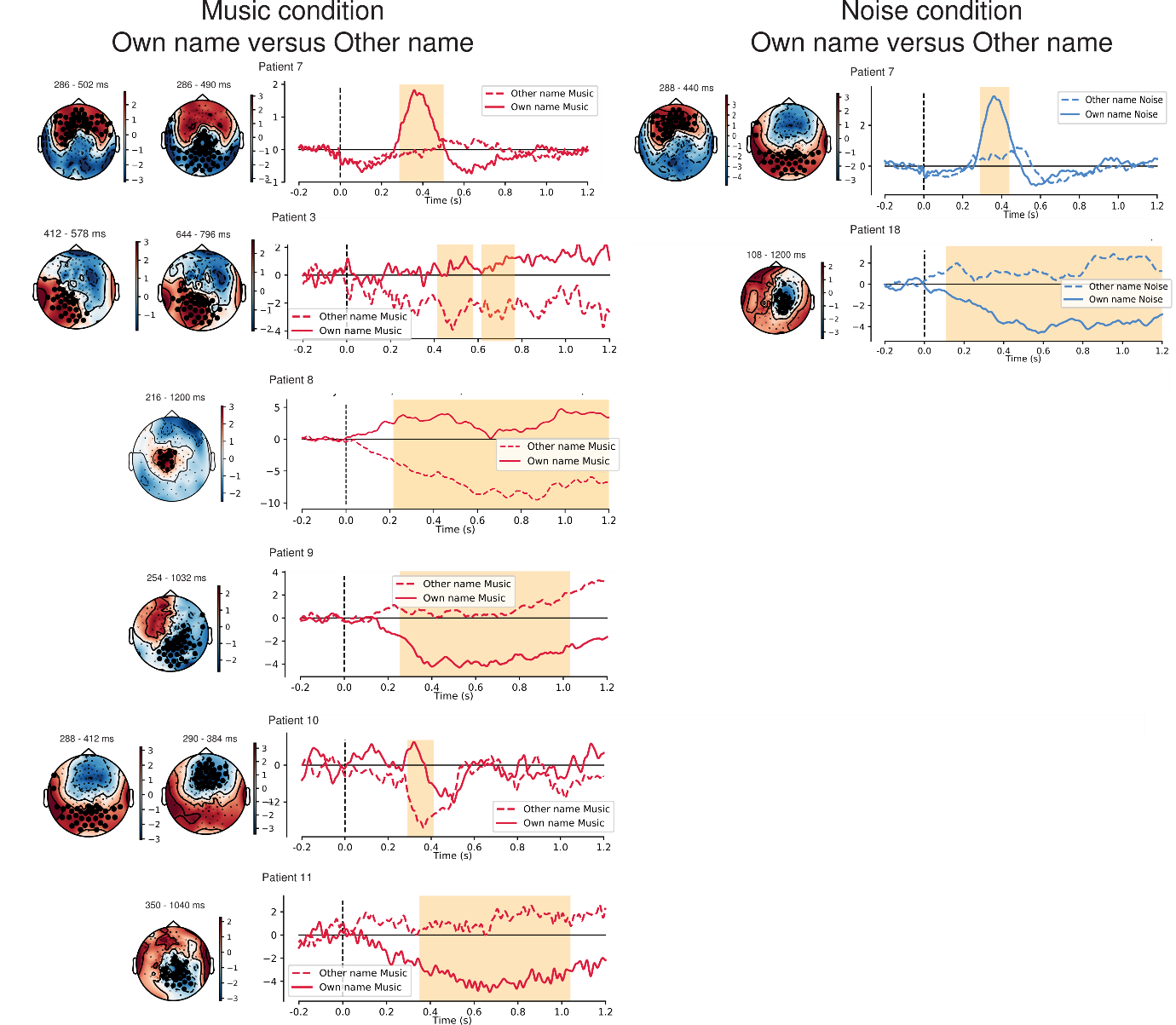


**Patients’ individual results within the music and noise conditions.**

Results of spatiotemporal cluster permutation tests within the patients. Significant clusters are indicated by black electrodes on each averaged map (within the timeframe of significance). ERP traces are averaged activity within significant clusters of electrodes, and significant timeframe is indicated with orange.
